# Impact of Electronic Transition and Prefilled Templates on Drug Prescription Compliance: Retrospective Study

**DOI:** 10.2196/57782

**Published:** 2025-04-09

**Authors:** Aurélien Lambert, Benoit Hombourger, Julia Salleron, Fadila Chergui, Catherine Vallance, Nadège Nicolas, Marie Moussouni, Lounisse Cherif, Emile Chenot, Céline Gavoille, Vincent Massard

**Affiliations:** 1 Institut de Cancérologie de Lorraine Vandoeuvre-lès-Nancy France; 2 Université de Lorraine, INSERM UMR 1319 INSPIIRE Vandoeuvre-lès-Nancy France

**Keywords:** drug prescription, electronic prescription, handwriting, medical oncology, ambulatory care

## Abstract

**Background:**

The transition from traditional handwritten prescriptions to electronic prescribing systems represents a significant advancement, with the potential to enhance treatment efficacy, patient safety, and professional communication.

**Objective:**

This study aimed to examine the impact of this transition within a medical oncology service, assessing the compliance of electronic prescriptions with established good practice standards and exploring the associated risks.

**Methods:**

In this retrospective analysis, we compared handwritten prescriptions from the pre-electronic era (January to May 2018) with electronic prescriptions (January to May 2021) following the implementation of the electronic prescribing system PandaLab Pro (PandaLab SAS). The inclusion criteria focused on outpatient oncology treatments, with a clear set of exclusion parameters to ensure a focused study scope. We defined good compliance as the written mention of the evaluated terms. The compliance rates were then compared using a chi-square test.

**Results:**

Our findings, based on a sample size of 260 prescriptions (randomized among 30,526 archived prescriptions), indicate a substantial improvement in electronic prescriptions’ compliance with prescribers and patient details, treatment accuracy, and overall adherence to regulatory standards. Notably, electronic formats achieved a remarkable 80.8% accuracy rate in compliance with safety criteria compared with 8.5% for handwritten prescriptions (*P*<.001). The use of prefilled prescriptions significantly increased compliance from a safety perspective (56% vs 96.2%; *P*<.001) compared with electronic prescriptions from scratch.

**Conclusions:**

The analysis further underscores the advantages of prefilled electronic prescription templates, which significantly improved compliance rates compared with manually filled electronic and handwritten prescriptions. Furthermore, the study revealed a marked shift in prescribing behaviors, with electronic prescriptions tending to be more concise yet more numerous, suggesting an impact on medication management and patient adherence, which warrants further investigation. The study supports the transition to electronic prescribing systems in oncology, highlighting enhanced traceability, compliance with health authority standards, and patient safety. The implementation of prefilled templates supported by pharmacists has emerged as a pivotal factor in this improved process. While acknowledging certain limitations, such as the nonquantitative assessment of time savings and acceptability, this research advocates for the widespread adoption of electronic prescriptions and serves as a benchmark for future e-prescription initiatives in France.

## Introduction

Drug prescriptions are a pivotal element in the care process and play a crucial role in treatment efficacy, patient safety, and communication among health care professionals. Traditionally, prescriptions were handwritten, a method prone to various errors and limitations; however, electronic prescriptions are not exempt from errors that can vary from 2 to 514 per 1000 prescriptions and from 4.2% to 82% of patients or charts reviewed [1]. With the advent of health information technologies, electronic prescriptions have emerged as a promising solution for enhancing care quality and medication treatment management. In oncology, in which therapeutic regimens are often complex and high-risk, the accuracy and clarity of prescriptions are particularly significant. The primary goal of electronic prescriptions is to minimize the risk of medication errors as much as possible while improving legibility [2,3].

Although the impact of introducing an electronic prescribing system on compliance with good prescribing practices in medical oncology services is evident, it has not been fully evaluated in oncology outpatients. However, electronic prescription introduces a new source of risk [4], one of which is called automation bias, which occurs when a physician blindly trusts prescription-helping software, thereby reducing vigilance in information-seeking and validation [5].

The transition from handwritten to electronic prescriptions in our center offers a unique opportunity to examine changes in prescription quality and their adherence to standards established by the authorities (Haute autorité de santé and Direction générale de l’offre de soins in France) [6].

Although historically ubiquitous, handwritten prescriptions are prone to errors owing to illegibility, omission of crucial information, and variability in interpreting instructions. These shortcomings can lead to prescription errors, which can affect patient safety and treatment efficacy. The time-consuming and redundant nature of handwritten prescriptions could also lead to shortcuts in writing or other “homegrown” systems of drafts, carbon papers, and Microsoft Word files in more or less secure folders. Not to mention the major identity-vigilance risks.

Oncology, which requires great precision in prescribing complex treatments, represents an ideal context for assessing the impact of prescription digitalization. Errors in prescribing antitumor agents or growth factors can have serious consequences, making the accuracy and clarity of information imperative. Furthermore, the digitization of prescriptions can also play a role in improving care coordination, a critical aspect of cancer treatment where multiple specialists are often involved in patient care. This study is part of a broader context of evolving health practices toward greater digitization, a change accelerated by the COVID-19 pandemic. The transition to more electronic health systems is seen not only as a means to improve operational efficiency but also as a crucial step to increase patient safety and care quality.

To conduct this study, we analyzed handwritten prescriptions from 2018, before the electronic era, and electronic prescriptions from 2021, after the deployment of our outpatient electronic prescribing solution. This comparative analysis allowed us to evaluate the evolution of several parameters, including the presence of essential information on prescribers and patients, adherence to prescription standards, and variety of prescribed treatments. Here, we report our center’s experience with the electronic transition of prescriptions, moving from entirely handwritten prescriptions to 100% electronic prescriptions 3 years later.

When dealing with the implications of prescribing medication for patient safety, it is necessary to consider 2 types of prescription criteria—the prescription writing process and the therapeutic decision. Prescription writing criteria include information about the patient, prescriber, and prescribed medications, considered during the prescription writing process. The therapeutic decision criteria determine the drugs selected by the prescriber to integrate the prescription document during their therapeutic decision process. For this study, prescription writing criteria were considered. Therefore, the main objective of the study was to assess the improvement in prescription compliance through an electronic solution compared with historical manual drafting.

## Methods

### Overview

We retrospectively collected prescriptions from our electronic patient records from January 1 to May 31, 2018 (2018 period), for handwritten prescriptions and from January 1 to May 31, 2021 (2021 period), for digitized prescriptions.

We selected prescriptions for outpatients in the oncology department to obtain the greatest possible completeness for different prescriptions. The prescription software we introduced, PandaLab Pro (PandaLab SAS), of course allows prescriptions from scratch, but above all, the use of prefilled prescription templates to save time and ensure a reproducible attitude, given that treatment protocols ultimately lead to many similarities in prescriptions (systematization of leukocyte growth factors associated with some chemotherapy regimens, biological tests planned in advance according to the protocol, and so on).

All prescription templates were created in advance by a team of medical oncologists and then underwent a thorough review by pharmacists to ensure compliance with the standards (Textbox 1).

The compliance criteria for the authorities are listed in Textbox 2.

It should be noted that a prescription was considered illegible if only 1 line was considered nonreadable, regardless of whether the rest was readable. Furthermore, 2 readers (1 oncologist and 1 pharmacist) performed the quality control of the readability of each prescription. A second joint reading was to be conducted in case of discrepancies.

We retrieved prescription types, such as antitumor agents or growth factors, and the rest were compiled in an associated treatment group.

**Inclusion and exclusion criteria.**

**Inclusion criteria:**

- All the patients underwent outpatient medical oncology treatment.
- Treatment prescription.
- Handwritten or electronic (no intermediate format).

**Exclusion criteria**:

- Prescription for nursing care (bandages, physiotherapy, and so on).
- Equipment (walking sticks, compression stockings, and so on).
- Simultaneous preprinted carbon and handwritten prescriptions.
- Chemotherapy prescriptions (intravenous molecules are prescribed in a specific chemotherapy software, but a paper copy is added to the patient’s file).
- Medical test prescriptions (laboratory work, imaging, and so on).

**Compliance criteria expected by the authorities.**

**Prescriber**

- First name (or first letter)
- Last name
- Specialty or unit
- Registration number (Répertoire Partagé des Professionnels de la Santé)
- Signature

**Center**

- Name
- Address
- Registration number (Fichier National des Établissements Sanitaires et Sociaux)

**General**

- Contact information (prescriber or center)
- Prescription date
- Readability

**Patient**

- First name
- Last name
- Birth date (or age)
- Social number

**Prescription type**

- Cancer treatment type (cancer treatment and associated treatment)

**Molecule**

- International drug name
- Commercial drug name
- Drug type (intravenous, oral, subcutaneous, and intramuscular)
- Dosage unit
- Drug dosage
- Posology
- Duration

### Assessment of Drug Delivery Methods

Concerning the international name of a molecule when several treatments were prescribed, it had to match every single molecule to be correct; if 1 or more names were incorrect (or with brand name only), the item was considered invalid.

For reproducibility and safety questions, we excluded specific prescriptions such as medical transport and exceptional molecules to maintain only routine prescriptions with molecules.

In 2018, all prescriptions were considered for inclusion and were randomly allocated using a randomization table to ensure unbiased assignment and to mitigate selection bias. We selected the first 130 patients who met the inclusion criteria. For the 2021 period, we were able to disqualify some prescriptions in advance because the type of prescription is now part of our file system (we excluded transport prescriptions, nursing and home care prescriptions, examinations, etc) The remaining prescriptions were then analyzed using the same logic as that for the 2018 period.

### Statistical Analysis

Statistical analysis was performed using the SAS software (SAS Institute Inc). Assuming a pessimistic hypothesis of 40% compliance in the case of handwritten prescriptions, we evaluated the potential of the electronic solution to improve this compliance rate to at least 60%, with a power of 90% and α risk of 5%. To test this hypothesis, it was necessary to conduct a 2-tailed *t* test to collect 130 prescriptions in each arm, making a total of 260 prescriptions. The significance level was set at 5%. Qualitative parameters of prescription characteristics and compliance rates were described by headcount and percentage, and quantitative parameters by median, and first and third quartiles. For the comparison of characteristics according to electronic formats and handwritten prescriptions, the quantitative parameters were compared using the Wilcoxon Mann-Whitney test, and the qualitative parameters were compared using the chi-square test. The compliance rates were compared using the chi-square test.

### Ethical Considerations

The internal scientific board of the Institut de Cancérologie de Lorraine approved this observational study, which was declared to the National Commission for Information Technology and Civil Liberties (CNIL) in France. It was registered as a standard declaration by the CNIL correspondent of the Cancer Institute of Lorraine (registration 111). They confirmed that all research was conducted in accordance with relevant guidelines, and informed consent was waived for all participants. All collected data were properly anonymized. No funding was required, and no compensation was provided to the patients for this study.

## Results

### Overview

In the handwritten arm, during the January-May 2018 period, a total of 251 prescriptions were analyzed from an initial pool of 6129 to keep the first 130 meeting the inclusion criteria.

In the electronic prescription arm, during the January-May 2021 period, out of 24,397 prescriptions, 8206 were filtered using electronic criteria, with only 150 prescriptions analyzed to keep the first 130 that met the inclusion criteria.

### Group Comparability

The study found a significant difference between the 2 groups regarding the presence of prefilled prescriptions (*P*<.001), with none in the handwritten group (Table 1). The handwritten group also had a significantly higher number of prescriptions for oral intake (*P*=.01). However, for critical criteria, such as the presence of antitumor agents, no significant difference was observed, indicating comparable quality in prescribing critical medications across both groups. It should be noted that 50% (65/130) of the prescriptions had 1-3 molecules in the handwritten group, while 50% (65/130) of the prescriptions had between 1 and 3 molecules in the electronic group.

**Table 1:** Prescription characteristics.

Characteristics	Handwritten (N=130), n (%)	Electronic (N=130), n (%)	*P* value
Prefilled	0 (0)	80 (61.5)	<.001
Antitumoral agent	40 (30.8)	32 (24.6)	.28
Growth factor	20 (15.4)	11 (8.5)	.09
Associated Treatment	92 (70.8)	94 (72.3)	.78
Oral	100 (76.9)	81 (62.3)	.01
Intramuscular or subcutaneous	37 (28.5)	40 (30.8)	.68
Intravenous	1 (0.8)	1 (0.8)	—^a^
Local	21 (16.2)	21 (16.2)	≥.99
Median molecule number (IQR)	1 (1-3)	1 (1-2)	<.001

^a^Not applicable.

### Compliance Analysis

Compliance analysis is shown in Table 2.

Compliance regarding the prescriber’s details was significantly higher in electronic prescriptions than in handwritten prescriptions (117/130, 90% vs 70/130, 53.8%; *P*=.03). This indicates a substantial improvement in ensuring that prescriber information is correctly included in the electronic formats.

Interestingly, the handwritten arm had excellent readability compared with the electronic arm (127/130, 97.7% vs 130/130, 100%; *P*=.08).

The compliance rate of patient identity information showed major differences, with 0% for handwritten prescriptions compared with 100% for electronic prescriptions. Even when the absence of a social security number was tolerated, handwritten prescriptions achieved a compliance rate of only 27.7%.

Treatment compliance, which refers to the correctness of the prescribed treatment details, also saw a significant improvement in electronic prescriptions over handwritten ones (82/130, 63.1% vs 6/130, 4.6%; *P*<.001), but more interestingly, when we tolerated the molecule to be presented with international or brand name indifferently (as it was a purely administrative criteria, not involving patient safety), the difference remained very significant (105/130, 80.8% vs 39/130, 30%; *P*<.001). If dosage, duration, or molecule name (international or brand) showed a good compliance of >80% each, the main difference was the absence or presence of the dosage unit in the handwritten and electronic groups, respectively, same goes for drug type; both items are very important concerning patient safety. This improvement indicates the higher reliability of electronic prescriptions for accurately conveying treatment information.

To be comparable, we had to test excluding the social number and birth date to reach an acceptable rate of compliance for the handwritten arm; however, the difference was still significant (105/130, 80.8% vs 130/130, 100%; *P*<.001).

The results clearly demonstrate the superior compliance of electronic prescriptions across all examined parameters. The digitization of prescriptions significantly enhances the accuracy of prescriber mentions, patient identity, and treatment details. Particularly noteworthy is the achievement of 100% accuracy in patient identity in electronic prescriptions, which is a critical factor for patient safety and treatment efficacy.

**Table 2:** Compliance analysis between handwritten and electronic groups.

Compliance items		Handwritten (N=130), n (%)	Electronic (N=130), n (%)	*P* value
**Prescriber**		70 (53.8)	117 (90)	.03
	Last name	124 (95.4)	130 (100)	.01
	First name	121 (93.1)	130 (100)	<.01
	Specialty or unit	120 (92.3)	117 (90)	.51
	Registration number	70 (53.8)	130 (100)	<.001
	Signature	127 (97.7)	130 (100)	.08
**Center**		130 (100)	130 (100)	—^a^
	Name	130 (100)	130 (100)	—
	Address	130 (100)	130 (100)	—
	Registration number	130 (100)	130 (100)	—
**Prescription**		65 (50)	112 (86.2)	<.001
	Contact	68 (52.3)	112 (86.2)	<.001
	Date	127 (97.7)	130 (100)	.08
	Readability	127 (97.7)	130 (100)	.08
	Prescription excluding contact^b^	124 (95.4)	130 (100)	.03
**Patient**		0 (0)	130 (100)	<.001
	Patient excluding social number^c^	36 (27.7)	130 (100)	<.001
	Patient excluding social number and birthdate^d^	105 (80.8)	130 (100)	<.001
	Last name	129 (99.2)	130 (100)	.32
	First name	105 (80.8)	130 (100)	<.001
	Birth date	36 (27.7)	130 (100)	<.001
	Social number	0 (0)	130 (100)	<.001
**Molecule (international name only)**		6 (4.6)	82 (63.1)	<.001
	Molecule (international or brand name)^e^	39 (30)	105 (80.8)	<.001
	International name	16 (12.3)	90 (69.2)	<.001
	Brand name	116 (89.2)	95 (73.1)	<.001
	Drug type	63 (48.5)	117 (90)	<.001
	Dosage unit	77 (59.2)	116 (89.2)	<.001
	Dosage	114 (87.7)	127 (97.7)	.002
	Duration	107 (82.3)	126 (96.9)	<.001

^a^Not applicable.

^b^Analysis was performed without considering the presence or absence of contact.

^c^Analysis was performed without considering the presence or absence of social number.

^d^Analysis was performed without considering the presence or absence of social number nor birthdate.

^e^Analysis was performed without considering the presence or absence of international name, brand name was allowed indifferently.

### Safety Versus Administrative Criteria

Considering all criteria indiscriminately, as presented in Table 3, compliance was significantly higher in the electronic group (62/130, 47.7% vs 0%; *P*<.001). However, this means that even this dedicated electronic software is under the threshold of 50% of prescriptions aligned with the expected results from the authorities.

Some items might be considered extremely difficult to follow or even useless from the clinician’s perspective. We try to discern what might really affect patient safety by considering three scenarios: (1) prescriptions without contact or social number but with strict international molecule names expected, (2) prescriptions without contact or social number and birth date but with strict international molecule names expected, and (3) tolerance of international and brand names. From a clinician’s perspective, the brand name does not endanger a patient; in France, it is more a matter of reimbursement and the possibility of substituting such molecules with generic or biosimilar treatments. Therefore, when focusing on patient safety criteria, those that could directly impact patient health, electronic prescriptions demonstrated superior compliance compared with handwritten prescriptions (105/130, 80.8% vs 11/130, 8.5%; *P*<.001). This significant discrepancy underscores the potential of electronic prescriptions in enhancing patient safety by reducing errors.

**Table 3:** Analysis considering safety or administrative criteria.

Compliance criteria		Handwritten (N=130), n (%)	Electronic (N=130), n (%)	*P* value
**Administrative criteria^a^**				
	All categories (prescriber, center, prescription, patient, and molecule)	0 (0)	62 (47.7)	<.001
**Patient safety criteria^b^**				
	Prescription excluding contact Patient excluding social number Molecule (international name allowed only)	1 (0.8)	82 (63.1)	<.001
	Prescription excluding contact Patient excluding social number and birthdate Molecule (international name allowed only)	4 (3.1)	82 (63.1)	<.001
	Prescription excluding contact Patient excluding social number Molecule (both international and brand name allowed)	11 (8.5)	105 (80.8)	<.001

^a^All criteria considered.

^b^Excluding criteria that do not directly endanger the patient.

### Prefilled or Not

The study further highlighted the benefits of using prefilled electronic prescription templates, which showed higher compliance rates than both manually filled electronic prescriptions and handwritten prescriptions (Table 4). With this in mind, prefilled prescriptions significantly increased compliance from all criteria (10/50, 20% vs 52/80, 65%; *P*<.001), but also from a safety perspective (28/50, 56% vs 77/80, 96.2%; *P*<.001).

**Table 4:** Analysis considering prefilled status in the electronic arm.

Compliance criteria		Not prefilled (N=50), n (%)	Prefilled (N=80), n (%)	*P* value
**Administrative criteria^a^**				
	All categories (prescriber, center, prescription, patient, and molecule)	10 (20)	52 (65)	<.001
**Patient safety criteria^b^**				
	Prescription excluding contact Patient excluding social number^c^ Molecule (international name allowed only)	26 (13)	69 (86.2)	<.001
	Prescription excluding contact Patient excluding social number Molecule (both international and brand name allowed)	28 (56)	77 (96.2)	<.001

^a^All criteria considered.

^b^Excluding criteria that do not directly endanger the patient.

^c^Birthdate was not considered as it was 100% present in both subgroups.

## Discussion

### Principal Findings

This study conducted a fair comparative analysis of electronic and handwritten prescription processes. Our findings reveal that electronic prescriptions have significantly improved the efficiency, accuracy, and safety of the prescription process compared with handwritten prescriptions. The accuracy of electronic prescriptions was significantly higher (105/130, 80.8% vs 11/130, 8.5%; *P*<.001) from a safety perspective. Even manual prescriptions (from scratch) were better in electronic format (28/50, 56%) than handwritten prescriptions (11/130, 8.5%).

These findings are consistent with 2 systematic reviews of the literature conducted by Mohsin-Shaik et al [7] and Osmani et al [8], where electronic prescriptions seem to favor safety and efficiency, but lack any solid proof to offer a consensus. Furthermore, electronic prescription solutions might introduce new challenges such as workflow disruptions and increased documentation time, while raising concerns about reliability and maintenance over time in a rapidly evolving digital environment [9].

In the handwritten group, prescriptions were analyzed from an initial pool of 6129 (compared with 24,397 in the electronic group), with 130 meeting the inclusion criteria due to the lack of a preselection filter. This period highlighted the challenges associated with manual prescription management, including exhaustive scanning and lower retention of prescriptions due to the nonelectronic nature of the process. This illustrates the lack of traceability and archiving of prescriptions before the electronic era.

The ease with which we were able to extract electronic prescriptions with the electronic system’s ability to categorize prescriptions (eg, by title, type, and medication) allowed for highly effective filtering, showcasing the advantage of electronic prescriptions over manual ones in terms of information retrieval and organization, is a sign of improved clinical practice.

The differentiation between full criteria compliance and selective safety criteria (excluding contact or nondecisive information) provides a nuanced understanding of the areas where electronic prescriptions excel, and handwritten prescriptions show strong gaps. We highlighted that some items might be considered extremely difficult to follow or even useless from the clinician’s perspective; this underscores the need for further studies to evaluate the practicality and clinical relevance of such criteria. Future studies should focus on identifying which criteria genuinely enhance patient safety and treatment accuracy, and which may be overly burdensome or redundant. By doing so, we can refine the electronic prescription process to better align with the realities of clinical practice, ultimately improving both compliance and efficiency. These studies could involve direct feedback from clinicians and a thorough analysis of the impact of each criterion on the quality of care and patient outcomes through standardized tools.

Few studies have been conducted on reducing serious medication errors by electronic prescribing [8,10]. This seems obvious but has not been fully validated. A well-conducted meta-analysis showed the positive effect of electronic solutions in reducing the risk of medication errors and side effects, notably assessing errors in terms of patient safety (not administrative accuracy) [8]. This study also showed a lack of quality literature and comparative studies in the field.

Yet, 1 study takes a contrary position by showing that other types of errors become possible and that additional criteria could improve the quality and safety of prescriptions, such as including the reason for prescribing a drug [11].

Furthermore, decreasing prescription errors does not necessarily lead to a reduction in patient harm [12].

In the comparative analysis of prescription modalities, our findings suggest a notable divergence between handwritten and electronic prescription behaviors. Specifically, manual prescriptions tend to aggregate a larger number of molecules, averaging between 1 and 3 per prescription, compared with electronic prescriptions, which typically feature 1-2 molecules. This discrepancy may be attributed to the inherently greater accessibility and availability of electronic prescriptions. The electronic platform’s capacity to store and manage a more extensive prescription repository, potentially capturing data not previously collected in manual formats, supports this observation.

Furthermore, the electronic prescription system’s usage of prefilled templates significantly streamlines the process of issuing multiple prescriptions. Practitioners are more likely to issue successive prescriptions for individual medications through these templates than to manually compile a single prescription for multiple drugs from scratch.

The tendency to prescribe fewer medications per electronic prescription, yet potentially more prescriptions overall, raises questions regarding the clinical significance of the prescription modality on medication management and patient adherence. Although the convenience and efficiency of electronic prescriptions are clear, the impact of this shift on patient outcomes, particularly in terms of medication adherence and the potential for increased health care interactions, warrants further investigation.

This also suggests that prefilled templates, reviewed by pharmacists, significantly contribute to prescription accuracy and should be encouraged, not to mention that blank prescriptions are also improving accuracy (28/50, 56%) compared with handwritten ones (11/130, 8.5%).

Pharmacists play a central role and should claim leadership in this field.

Another role they might endorse is the development of new tools to identify medication errors, wrong patient order entries, or even unnecessary prescriptions, as presented by Garrod et al [13] in their scoping review, potentially aided by artificial intelligence one day.

Concerning the old handwritten process, prescriptions were digitized in the document management system but required the physician’s voluntary action; if a duplicate was given to the patient, there was no follow-up. It is very difficult to assess the lack of traceability, but it is probably very significant, given the data we have observed since the 100% traceable solution. With the software, it is still possible to make a manual free prescription without directly interfacing the patient’s data, but this is a negligible operation.

Interestingly, handwritten prescriptions were considered readable in 97.7% (127/130) of cases (130/130, 100% in the electronic arm), which contradicts the long-held belief that physicians write poorly.

The historical comparison of electronic and handwritten prescriptions, from a period devoid of electronic tools to an era with a fully deployed electronic system, was pivotal. We intentionally selected comparable periods to ensure the validity of our comparison, mindful of the seasonal variations in prescription habits.

Although we did not evaluate the time required for each prescription method, as it would not have been easily reproducible, our qualitative observations suggest that electronic prescriptions save time and reduce complexity. A survey not planned when the prescription system was set up found a satisfaction rate of 88.2% among the 11 responding physicians. However, there is an important distinction between different types of electronic solutions. Generalist software, like many electronic health records that are not dedicated to prescriptions, can be unwelcomed and have been associated with increased practitioner burnout [14]. By contrast, a tailor-made system that integrates patient data and facilitates order entry can make a substantial difference. All comes to computerized physician order entry, which is fundamentally the major difference between 2 so-called “electronic software.” A read-only patient’s file is very different from an electronic health record with computerized physician order entries from the burnout point of view.

We believe that our approach is perfectly aligned with the upcoming deployment of e-prescriptions in France, which will overcome certain limitations.

### Conclusion

Our study focuses on the specific practice of electronic prescribing in a context that is extremely demanding in terms of time consumption and complexity in oncology. This practice is often confused with the computerization of patient records but deals with entirely different issues and acceptability by health care professionals.

Our findings support a transition to electronic prescriptions, which are not only more numerous, but also provide better traceability, compliance with health authority standards, and patient safety. The use of prefilled templates shows great promise, underscoring the pharmacist’s critical role in the prescription process.

While the study boasts a high readability score and significant improvement in prescription accuracy with electronic solutions, we recognize certain limitations. Acceptability and the actual time saved were not quantitatively assessed, and these aspects could be explored in future multicenter studies to minimize center bias.

In conclusion, the transition to electronic prescriptions represents a leap forward in the quality of the prescription process. The integration of prefilled templates and the central role of pharmacists are key factors in this improvement. The compliance of prescriptions with all combined criteria improved from 8.5% (11/130) to 47.7% (62/130) through electronic means, and even reached up to 80.8% (105/130) when considering patient safety criteria. This study advocates the widespread adoption of prefilled electronic prescription templates to further enhance prescription quality and patient safety. Our study also serves as a comparative benchmark for other software solutions aimed at demonstrating enhancements and contributing to long-term support of the e-prescription movement in France.

## Abbreviations

CNIL: National Commission for Information Technology and Civil Liberties

## References

[1] Ross S, Bond C, Rothnie H, Thomas S, Macleod MJ (2009). What is the scale of prescribing errors committed by junior doctors? A systematic review. Br J Clin Pharmacol.

[2] Aronson JK (2009). Medication errors: what they are, how they happen, and how to avoid them. QJM.

[3] Hitti E, Tamim H, Bakhti R, Zebian D, Mufarrij A (2017). Impact of internally developed electronic prescription on prescribing errors at discharge from the emergency department. West J Emerg Med.

[4] Alshahrani F, Marriott JF, Cox AR (2021). A qualitative study of prescribing errors among multi-professional prescribers within an e-prescribing system. Int J Clin Pharm.

[5] Lyell D, Magrabi F, Raban MZ, Pont LG, Baysari MT, Day RO, Coiera E (2017). Automation bias in electronic prescribing. BMC Med Inform Decis Mak.

[6] Arrêté du 31 mars 1999 relatif à la prescription, à la dispensation et à l'administration des médicaments soumis à la réglementation des substances vénéneuses dans les établissements de santé, les syndicats interhospitaliers et les établissements médico-sociaux disposant d'une pharmacie à usage intérieur mentionnés à l'article L. 595-1 du code de la santé publique. Légifrance.

[7] Mohsin-Shaikh S, Furniss D, Blandford A, McLeod M, Ma T, Beykloo MY, Franklin BD (2019). The impact of electronic prescribing systems on healthcare professionals' working practices in the hospital setting: a systematic review and narrative synthesis. BMC Health Serv Res.

[8] Osmani F, Arab-Zozani M, Shahali Z, Lotfi F (2023). Evaluation of the effectiveness of electronic prescription in reducing medical and medical errors (systematic review study). Ann Pharm Fr.

[9] Van Wilder A, Bell H, Franklin BD (2016). The effect of electronic prescribing and medication administration on nurses’ workflow and activities: an uncontrolled before and after study. Safety in Health.

[10] Rosa MB, Nascimento MMGD, Cirilio PB, Santos RDA, Batista LF, Perini E, Couto RC (2019). Electronic prescription: frequency and severity of medication errors. Rev Assoc Med Bras (1992).

[11] Schiff G, Mirica MM, Dhavle AA, Galanter WL, Lambert B, Wright A (2018). A prescription for enhancing electronic prescribing safety. Health Aff (Millwood).

[12] van Doormaal FF, Di Nisio M, Otten H-M, Richel DJ, Prins M, Buller HR (2011). Randomized trial of the effect of the low molecular weight heparin nadroparin on survival in patients with cancer. J Clin Oncol.

[13] Garrod M, Fox A, Rutter P (2023). Automated search methods for identifying wrong patient order entry-a scoping review. JAMIA Open.

[14] Shanafelt TD, Dyrbye LN, Sinsky C, Hasan O, Satele D, Sloan J, West CP (2016). Relationship between clerical burden and characteristics of the electronic environment with physician burnout and professional satisfaction. Mayo Clin Proc.

